# Supplementary material for: Melatonin: The smart molecule that differentially modulates autophagy in tumor and normal placental cells
Source: PLoS One. 2019 Jan 10;14(1):e0202458. doi: 10.1371/journal.pone.0202458 (PMC6328125; doi:10.1371/journal.pone.0202458)
Supplement: S1 File — Raw data of all of the results presented in this manuscript. (DOCX) [file pone.0202458.s001.docx]

**Manuscript entitled:** Melatonin: the smart molecule that differentially modulates autophagy in tumor and normal placental cells

### Raw data files and statistical analysis used for generation of Figs 1 – 6

**BeWo viability measurement (Fig. 1b)**

|  | Normoxia | | | H/R | | |
| --- | --- | --- | --- | --- | --- | --- |
| Group | Average | Std dev | N | Average | Std dev | N |
| DMSO | 100.000000 | 12.500000 | 9 | 72.873270 | 9.766103 | 9 |
| MEL 1mM | 82.051930 | 7.320060 | 9 | 59.864520 | 4.590479 | 9 |
| RAP | 104.449600 | 7.148348 | 9 | 92.661190 | 6.934567 | 7 |
| 3-MA | 101.248900 | 9.644550 | 9 | 69.563930 | 4.319417 | 7 |

| Parameter |  |  |  |  |
| --- | --- | --- | --- | --- |
| Table Analyzed | Data 1 |  |  |  |
| One-way ANOVA |  |  |  |  |
|  |  |  |  |  |
| Source of Variation | % of total variation | P value |  |  |
| Interaction | 4.47 | 0.0072 |  |  |
| Column factor | 43.99 | < 0.0001 |  |  |
| Row factor | 31.28 | < 0.0001 |  |  |
|  |  |  |  |  |
| Source of Variation | P value summary | Significant? |  |  |
| Interaction | ** | Yes |  |  |
| Column factor | *** | Yes |  |  |
| Row factor | *** | Yes |  |  |
|  |  |  |  |  |
| Source of Variation | Df | Sum-of-squares | Mean square | F |
| Interaction | 3 | 918.4 | 306.1 | 4.411 |
| Column factor | 1 | 9040 | 9040 | 130.3 |
| Row factor | 3 | 6429 | 2143 | 30.88 |
| Residual | 60 | 4164 | 69.39 |  |
|  |  |  |  |  |
| Student’s *t*-test |  |  |  |  |
| Normoxia vs H/R |  |  |  |  |
| Row factor | Normoxia | H/R | Difference | 95% CI of diff. |
| DM | 100.0 | 72.87 | -27.13 | -37.24 to -17.01 |
| MEL 1 | 82.05 | 59.86 | -22.19 | -32.30 to -12.07 |
| R | 104.4 | 92.66 | -11.79 | -22.60 to -0.9774 |
| 3- | 101.2 | 69.56 | -31.68 | -42.50 to -20.87 |
|  |  |  |  |  |
| Row factor | Difference | t | P value | Summary |
| DMSO | -27.13 | 6.908 | P<0.01 | ** |
| MEL 1mM | -22.19 | 5.650 | P<0.01 | ** |
| RAP | -11.79 | 2.808 | P < 0.05 | ^#^, * |
| 3-MA | -31.68 | 7.547 | P<0.01 | ** |

**BeWo HCG secretion (Fig. 1c)**

|  | Normoxia | | | H/R | | |
| --- | --- | --- | --- | --- | --- | --- |
| Group | Average | Std dev | N | Average | Std dev | N |
| DMSO | 100.000000 | 8.281730 | 5 | 72.873270 | 14.272600 | 4 |
| MEL | 83.132580 | 11.435730 | 5 | 70.363710 | 9.230582 | 4 |
| RAP | 61.672200 | 15.093210 | 5 | 68.064300 | 18.019990 | 4 |
| 3-MA | 108.070900 | 9.920430 | 5 | 53.955200 | 9.936420 | 4 |

| Table Analyzed | Data 1 |  |  |  |
| --- | --- | --- | --- | --- |
|  |  |  |  |  |
| One way-ANOVA |  |  |  |  |
|  |  |  |  |  |
| Source of Variation | % of total variation | P value |  |  |
| Interaction | 28.73 | 0.0002 |  |  |
| Column factor | 28.29 | < 0.0001 |  |  |
| Row factor | 14.86 | 0.0071 |  |  |
|  |  |  |  |  |
| Source of Variation | P value summary | Significant? |  |  |
| Interaction | *** | Yes |  |  |
| Column factor | *** | Yes |  |  |
| Row factor | ** | Yes |  |  |
|  |  |  |  |  |
| Source of Variation | Df | Sum-of-squares | Mean square | F |
| Interaction | 3 | 4331 | 1444 | 9.535 |
| Column factor | 1 | 4265 | 4265 | 28.17 |
| Row factor | 3 | 2241 | 747.0 | 4.934 |
| Residual | 28 | 4239 | 151.4 |  |
|  |  |  |  |  |
| Student’s *t*-test |  |  |  |  |
|  |  |  |  |  |
| Normoxia vs H/R |  |  |  |  |
| Row factor | Normoxia | H/R | Difference | 95% CI of diff. |
| DMSO | 100.0 | 72.87 | -27.13 | -49.16 to -5.092 |
| MEL | 83.13 | 70.36 | -12.77 | -34.80 to 9.266 |
| RAP | 61.67 | 68.06 | 6.392 | -15.64 to 28.43 |
| 3-MA | 108.1 | 53.96 | -54.12 | -76.15 to -32.08 |
|  |  |  |  |  |
| Row factor | Difference | t | P value | Summary |
| DMSO | -27.13 | 3.286 | P < 0.05 | * |
| MEL | -12.77 | 1.547 | P > 0.05 | ns |
| RAP | 6.392 | 0.7744 | P > 0.05 | ns |
| 3-MA | -54.12 | 6.556 | P<0.01 | ** |

**BeWo expression of Phospho-AMPK/Total AMPK (Fig. 2A)**

| Normoxia | | H/R | |
| --- | --- | --- | --- |
| DMSO | MEL | DMSO | MEL |
| 161.00000 | 768.24690 | 1419.707 | 768.069 |
| 100.00000 | 553.02540 | 1020.191 | 1942.941 |
| 100.00000 | 693.02770 | 1262.880 | 2178.310 |
| \| 115.00000 \| \| --- \| | 684.39550 | 1080.15620 | 1614.94150 |

| One-way analysis of variance |  |  |  |  |
| --- | --- | --- | --- | --- |
| P value | 0.0070 |  |  |  |
| P value summary | ** |  |  |  |
| Are means signif. different? (P < 0.05) | Yes |  |  |  |
| Number of groups | 4 |  |  |  |
| F | 8.577 |  |  |  |
| R squared | 0.7628 |  |  |  |
|  |  |  |  |  |
| ANOVA Table | SS | df | MS |  |
| Treatment (between columns) | 4009000 | 3 | 1336000 |  |
| Residual (within columns) | 1246000 | 8 | 155800 |  |
| Total | 5255000 | 11 |  |  |
|  |  |  |  |  |
| Newman-Keuls Multiple Comparison Test | Mean Diff. | q | P < 0.05? | Summary |
| DMSO vs MEL | -1530 | 6.713 | Yes | *** |
| DMSO vs DMSO | -1134 | 4.977 | Yes | *** |
| DMSO vs MEL | -571.4 | 2.508 | Yes | * |
| MEL vs MEL | -958.3 | 4.205 | Yes | *** |

**BeWo expression of Phospho-PP2Ac/Total PP2Ac (Fig. 2B)**

| Normoxia | | H/R | |
| --- | --- | --- | --- |
| DMSO | MEL | DMSO | MEL |
| 100.00000 | 184.39550 | 69.24640 | 66.1707 |
| 100.00000 | 84.90611 | 88.47250 | 83.3254 |
| 100.00000 | 116.69590 | 19.85130 | 57.7634 |
| 100.00000 | 60.89893 | 36.94590 | 26.0109 |

| Table Analyzed | Data 1 |  |  |  |
| --- | --- | --- | --- | --- |
|  |  |  |  |  |
| One-way analysis of variance |  |  |  |  |
| P value | 0.0349 |  |  |  |
| P value summary | * |  |  |  |
| Are means signif. different? (P < 0.05) | Yes |  |  |  |
| Number of groups | 4 |  |  |  |
| F | 2.670 |  |  |  |
| R squared | 0.4003 |  |  |  |
|  |  |  |  |  |
| ANOVA Table | SS | df | MS |  |
| Treatment (between columns) | 133800 | 3 | 44610 |  |
| Residual (within columns) | 200500 | 12 | 16710 |  |
| Total | 334300 | 15 |  |  |
|  |  |  |  |  |
| Newman-Keuls Multiple Comparison Test | Mean Diff. | q | Significant? P < 0.05? | Summary |
| MEL vs MEL | -241.6 | 6.713 | Yes | * |
| DMSO vs DMSO | -261.9 | 4.977 | Yes | * |
| MEL vs DMSO | -2.026 | 2.508 | No | ns |
| DMSO vs MEL | -199.6 | 4.205 | No | ns |

**BeWo expression of Beclin-1 (Fig. 2C)**

| Normoxia | | | H/R | | |
| --- | --- | --- | --- | --- | --- |
| DMSO | MEL | RAP | DMSO | MEL | RAP |
| 100. | 72.19473 | 185.4052 | 235.09070 | 196.74650 | 372.19470 |
| 100. | 137.27550 | 265.5019 | 187.45670 | 177.75310 | 325.40520 |
| 100. | 155.64900 | 254.8342 | 211.75390 | 133.75220 | 312.53290 |
| 100. | 77.62914 |  | 220.76430 | 138.85370 |  |
| 100. | 147.44380 |  |  | 136.98770 |  |

| One-way analysis of variance |  |  |  |  |  |
| --- | --- | --- | --- | --- | --- |
| P value | < 0.0001 |  |  |  |  |
| P value summary | *** |  |  |  |  |
| Means signif. different? (P < 0.05) | Yes |  |  |  |  |
| Number of groups | 6 |  |  |  |  |
| F | 30.49 |  |  |  |  |
| R squared | 0.8892 |  |  |  |  |
|  |  |  |  |  |  |
| ANOVA Table | SS | df | MS |  |  |
| Treatment (between columns) | 137400 | 5 | 27470 |  |  |
| Residual (within columns) | 17120 | 19 | 901.0 |  |  |
| Total | 154500 | 24 |  |  |  |
|  |  |  |  |  |  |
| Newman-Keuls Multiple Comparison Test | Mean Diff. | q | Significant? P < 0.05? | Summary | 95% CI of diff |
| DMSO vs MEL | -13.04 | 0.9713 | No | ns | -73.03 to 46.95 |
| DMSO vs DMSO | -108.8 | 7.639 | Yes | *** | -172.4 to -45.14 |
| DMSO vs MEL | -51.82 | 3.860 | No | ns | -111.8 to 8.173 |
| MEL vs DMSO | -95.73 | 6.723 | Yes | ** | -159.4 to -32.10 |
| MEL vs MEL | -38.78 | 2.889 | No | ns | -98.77 to 21.21 |
| MEL vs RAP | -218.7 | 14.11 | Yes | *** | -287.9 to -149.4 |
| RAP vs DMSO | 21.48 | 1.325 | No | ns | -50.97 to 93.93 |
| RAP vs MEL | 78.43 | 5.060 | Yes | *** | 9.156 to 147.7 |
| RAP vs RAP | -101.5 | 5.855 | Yes | ** | -178.9 to -24.02 |
| DMSO vs MEL | 56.95 | 4.000 | No | ns | -6.683 to 120.6 |
| DMSO vs RAP | -122.9 | 7.584 | Yes | *** | -195.4 to -50.50 |
| MEL vs RAP | -179.9 | 11.61 | Yes | *** | -249.2 to -110.6 |

**BeWo protein content of ATG7 (Fig. 3B)**

| Normoxia | | | H/R | | |
| --- | --- | --- | --- | --- | --- |
| DMSO | MEL | RAP | DMSO | MEL | RAP |
| 100. | 177.87890 | 250.4597 | 237.4819 | 362.00850 | 465.833800 |
| 100. | 103.32250 |  | 204.5394 | 343.50370 |  |
| 100. | 203.35260 |  | 151.0475 | 320.78190 |  |
| 100. | 145.46940 |  | 233.0573 | 346.86170 |  |
| 100. | 144.46480 |  | 242.9475 | 376.31620 |  |
| 100. | 155.38240 |  | 165.8373 | 374.46480 |  |

| One-way analysis of variance |  |  |  |  |
| --- | --- | --- | --- | --- |
| P value | < 0.0001 |  |  |  |
| P value summary | *** |  |  |  |
| Are means signif. different? (P < 0.05) | Yes |  |  |  |
| Number of groups | 4 |  |  |  |
| P value | 90.21 |  |  |  |
| One-way analysis of variance | 0.9312 |  |  |  |
|  |  |  |  |  |
| P value | 0.0410 |  |  |  |
| P value summary | * |  |  |  |
| Do the variances differ signif. (P < 0.05) | Yes |  |  |  |
|  |  |  |  |  |
| ANOVA Table | SS | df | MS |  |
| Treatment (between columns) | 218500 | 3 | 72840 |  |
| Residual (within columns) | 16150 | 20 | 807.5 |  |
| Total | 234700 | 23 |  |  |
|  |  |  |  |  |
| Newman-Keuls Multiple Comparison Test | Mean Diff. | q | Significant? P < 0.05? | Summary |
| DMSO vs MEL | -257.3 | 22.18 | Yes | *** |
| DMSO vs DMSO | -109.2 | 9.410 | Yes | *** |
| DMSO vs MEL | -58.32 | 5.028 | Yes | ** |
| MEL vs MEL | -199.0 | 17.16 | Yes | *** |
| MEL vs DMSO | -50.84 | 4.383 | Yes | ** |
| DMSO vs MEL | -148.2 | 12.77 | Yes | *** |

**BeWo LC3B-II protein content (Figure 3c)**

| Normoxia | | | H/R | | |
| --- | --- | --- | --- | --- | --- |
| DMSO | MEL | RAP | DMSO | MEL | RAP |
| 100.00000 | 11.93300 | 483.582200 | 242.323300 | 386.37500 | 617.85540 |
| 100.00000 | 137.09950 |  | 147.148900 | 196.13620 |  |
| 100.00000 | 64.82899 |  | 208.212600 | 156.27960 |  |
| 106.60000 | 190.70550 |  | 305.027700 | 212.20170 |  |
| 23.56928 | 90.39797 |  | 486.107600 | 96.39732 |  |
| 218.92110 | 87.92138 |  | 35.003300 | 98.99800 |  |

| One-way analysis of variance |  |  |  |  |
| --- | --- | --- | --- | --- |
| P value | 0.042074 |  |  |  |
| P value summary | * |  |  |  |
| Are means signif. different? (P < 0.05) | Yes |  |  |  |
| Number of groups | 4 |  |  |  |
| F | 1.573 |  |  |  |
| R squared | 0.2548 |  |  |  |
|  |  |  |  |  |
| ANOVA Table | SS | df | MS |  |
| Treatment (between columns) | 93230 | 5 | 18650 |  |
| Residual (within columns) | 272700 | 23 | 11860 |  |
| Total | 366000 | 28 |  |  |
|  |  |  |  |  |
| Newman-Keuls Multiple Comparison Test | Mean Diff. | q | Significant? P < 0.05? | Summary |
| DMSO N vs MEL H/R | -123.4 | --- | No | ns |
| DMSO N vs DMSO H/R | -151.7 | 6.726273 | No | ns |
| DMSO N vs MEL N | -80.96 | --- | No | ns |
| MEL N vs MEL H/R | -42.48 | --- | No | ns |
| MEL N vs DMSO H/R | -30.77 | --- | No | ns |

**BeWo Protein expression of P62 in H/R conditions (Fig 4a)**

| H/R | | | |
| --- | --- | --- | --- |
| No BAF A1 | | BAF A1 10nM | |
| DMSO | MEL | DMSO | MEL |
| 77.7253 | 111.7384 | 726.51350 | 306.7260 |
| 86.1010 | 126.7861 | 603.56410 | 254.3837 |
| 113.2395 | 114.9460 | 402.16380 | 422.3635 |
| 19.8947 | 225.1472 | 860.99500 | 201.1928 |
| 55.9840 | 369.4043 |  |  |

| Table Analyzed | Data 1 |  |  |  |
| --- | --- | --- | --- | --- |
|  |  |  |  |  |
| One-way analysis of variance |  |  |  |  |
| P value | < 0.001 |  |  |  |
| P value summary | ** |  |  |  |
| Are means signif. different? (P < 0.05) | Yes |  |  |  |
| Number of groups | 4 |  |  |  |
| F | 19.25 |  |  |  |
| R squared | 0.8049 |  |  |  |
|  |  |  |  |  |
| ANOVA Table | SS | df | MS |  |
| Treatment (between columns) | 803700 | 3 | 267900 |  |
| Residual (within columns) | 194800 | 14 | 13920 |  |
| Total | 998500 | 17 |  |  |
|  |  |  |  |  |
| Newman-Keuls Multiple Comparison Test | Mean Diff. | q | Significant? P < 0.05? | Summary |
| DMSO vs DMSO | -577.7 | 10.32 | Yes | ** |
| DMSO vs MEL | -225.6 | 4.031 | Yes | * |
| DMSO vs MEL | -119.0 | 2.256 | No | ns |
| MEL vs MEL | -106.6 | 1.904 | No | ns |

**BeWo Protein expression of Nrf2 (Fig 4b)**

| Normoxia | | | H/R | |
| --- | --- | --- | --- | --- |
| DMSO | MEL | SFN 10 μM | DMSO | MEL |
| 150.00000 | 42.83741 | 116.8235 | 314.3438 | 269.65500 |
| 85.00000 | 65.88973 | 242.7504 | 242.7504 | 107.25200 |
| 120.00000 | 89.85131 | 445.6733 | 306.0482 | 193.85930 |
| 100.00000 | 108.53870 | 352.3331 | 156.3648 | 72.23429 |
| 80.00000 |  |  | 314.4300 |  |

| One-way analysis of variance |  |  |  |  |
| --- | --- | --- | --- | --- |
| P value | 0.0057 |  |  |  |
| P value summary | ** |  |  |  |
| Are means signif. different? (P < 0.05) | Yes |  |  |  |
| Number of groups | 5 |  |  |  |
| F | 5.345 |  |  |  |
| R squared | 0.5571 |  |  |  |
|  |  |  |  |  |
| ANOVA Table | SS | df | MS |  |
| Treatment (between columns) | 154300 | 4 | 38580 |  |
| Residual (within columns) | 122700 | 17 | 7217 |  |
| Total | 277000 | 21 |  |  |
|  |  |  |  |  |
| Newman-Keuls Multiple Comparison Test | Mean Diff. | q | Significant? P < 0.05? | Summary |
| MEL vs DMSO | -190.0 | 4.715 | Yes | * |
| MEL vs MEL | -109.0 | 2.565 | No | ns |
| MEL vs DMSO | -30.22 | --- | No | ns |
| DMSO vs DMSO | -159.8 | 4.206 | Yes | * |
| DMSO vs MEL | -78.75 | --- | No | ns |
| MEL vs DMSO | -81.04 | --- | No | ns |

**BeWo Protein expression of Cleaved PARP-1/PARP-1 (Fig 4c)**

| Normoxia | | | H/R | | |
| --- | --- | --- | --- | --- | --- |
| DMSO | MEL | RAP | DMSO | MEL | RAP |
| 100.000000 | 91.41441 | 62.801240 | 66.87990 | 180.64420 | 174.83400 |
| 100.000000 | 199.50970 | 211.514600 | 338.53260 | 120.19050 | 95.45602 |
| 100.000000 | 300.94960 | 3.490018 | 287.26050 | 519.35900 | 287.01450 |
| 100.000000 | 181.51460 | 62.801240 | 332.97430 | 453.09430 | 195.06500 |
| 100.000000 | 95.90380 | 25.812400 | 55.98500 | 333.95300 | 120.08500 |

| Table Analyzed | Data 1 |  |  |  |
| --- | --- | --- | --- | --- |
|  |  |  |  |  |
| One-way analysis of variance |  |  |  |  |
| P value | 0.0367 |  |  |  |
| P value summary | ns |  |  |  |
| Are means signif. different? (P < 0.05) | No |  |  |  |
| Number of groups | 4 |  |  |  |
| F | 3.094 |  |  |  |
| R squared | 0.3671 |  |  |  |
|  |  |  |  |  |
| Bartlett's test for equal variances |  |  |  |  |
| Bartlett's statistic (corrected) | 12.62 |  |  |  |
| P value | 0.0055 |  |  |  |
| P value summary | ** |  |  |  |
| Do the variances differ signif. (P < 0.05) | Yes |  |  |  |
|  |  |  |  |  |
| ANOVA Table | SS | df | MS |  |
| Treatment (between columns) | 133100 | 3 | 44380 |  |
| Residual (within columns) | 229500 | 16 | 14340 |  |
| Total | 362700 | 19 |  |  |
|  |  |  |  |  |
| Newman-Keuls Multiple Comparison Test | Mean Diff. | q | Significant? P < 0.05? | Summary |
| DMSO vs MEL | -226.0 | 4.220 | Yes | * |
| DMSO vs DMSO | -220.9 | 4.258 | Yes | * |
| DMSO vs MEL | -278.46 | 5.736 | Yes | * |
| MEL vs MEL | -147.6 | 2.755 | No | ns |
| MEL vs DMSO | -42.47 | --- | No | ns |
| DMSO vs MEL | -105.1 | --- | No | ns |

**BeWo Protein expression of Nrf2 (Nuclear and cytosolic fractions)**

**(Fig 4d)**

| H/R | | | |
| --- | --- | --- | --- |
| DMSO | | MEL | |
| Nucleus | Cyto | Nucleus | Cyto |
| 2986.68400 | 133.06750 | 2226.4710 | 123.68240 |
| 2293.20000 | 171.38590 | 400.7336 | 113.98630 |
| 5263.56000 | 188.23100 | 700.9383 | 615.38370 |
| 2373.94800 | 154.83830 | 1000.8370 | 145.83730 |

| Table Analyzed | Data 1 |
| --- | --- |
| Column Nucleus DMSO | Nucleus |
| vs | vs |
| Column Nucleus Melatonin | Nucleus |
|  |  |
| Unpaired t test |  |
| P value | < 0.0001 |
| P value summary | *** |
| Are means signif. different? (P < 0.05) | Yes |
| One- or two-tailed P value? | Two-tailed |
| t, df | t=2.675 df=6 |
|  |  |
| How big is the difference? |  |
| Mean ± SEM of column A | 3229 ± 395.5 N=4 |
| Mean ± SEM of column C | 1082 ± 400.6 N=4 |
| Difference between means | 2147 ± 802.6 |
| 95% confidence interval | 183.0 to 4111 |
| R squared | 0.5439 |
|  |  |
| F test to compare variances |  |
| F,DFn, Dfd | 3.014, 3, 3 |
| P value | 0.3890 |
| P value summary | ns |
| Are variances significantly different? | No |

**BeWo Protein expression of TOM20 (Fig. 5a)**

| Normoxia | | H/R | | |
| --- | --- | --- | --- | --- |
| DMSO | MEL | DMSO | MEL | BAF A1 |
| 100. | 59.93741 | 26.17530 | 4.43680 | 94.40770 |
| 100. | 76.30316 | 57.49330 | 40.46980 |  |
| 100. | 46.39102 | 38.05123 | 21.72150 |  |
| 100. | 39.93741 | 26.17530 | 4.43680 |  |
| 100. | 46.30316 | 57.49330 | 40.46980 |  |
| 100. | 56.39102 | 38.05123 | 21.72150 |  |

| Parameter |  |  |  |  |
| --- | --- | --- | --- | --- |
| Table Analyzed | Data 1 |  |  |  |
|  |  |  |  |  |
| One-way analysis of variance |  |  |  |  |
| P value | < 0.0263 |  |  |  |
| P value summary | * |  |  |  |
| Are means signif. different? (P < 0.05) | Yes |  |  |  |
| Number of groups | 4 |  |  |  |
| F | 41.96 |  |  |  |
| R squared | 0.8629 |  |  |  |
|  |  |  |  |  |
| Bartlett's test for equal variances |  |  |  |  |
| Bartlett's statistic (corrected) |  |  |  |  |
| P value |  |  |  |  |
| P value summary | ns |  |  |  |
| Do the variances differ signif. (P < 0.05) | No |  |  |  |
|  |  |  |  |  |
| ANOVA Table | SS | df | MS |  |
| Treatment (between columns) | 19840 | 3 | 6613 |  |
| Residual (within columns) | 3152 | 20 | 157.6 |  |
| Total | 22990 | 23 |  |  |
|  |  |  |  |  |
| Newman-Keuls Multiple Comparison Test | Mean Diff. | q | Significant? P < 0.05? | Summary |
| MEL vs DMSO | -77.79 | 15.18 | Yes | * |
| MEL vs MEL | -32.00 | 6.244 | Yes | * |
| MEL vs DMSO | -18.36 | 3.583 | Yes | * |
| DMSO vs DMSO | -59.43 | 11.60 | Yes | * |
| DMSO vs MEL | -13.64 | 2.661 | No | ns |
| MEL vs DMSO | -45.79 | 8.934 | Yes | * |

**Human primary villous cytotrophoblast Nrf2 expression (Fig. 6b)**

| Normoxia | | H/R | |
| --- | --- | --- | --- |
| DMSO | MEL | DMSO | MEL |
| 100.00000 | 42.83741 | 314.3438 | 269.65500 |
| 100.00000 | 65.88973 | 242.7504 | 307.25200 |
| 100.00000 | 89.85131 | 306.0482 | 393.85930 |
| 100.00000 | 108.53870 | 156.3648 | 572.23430 |
|  |  | 314.4300 |  |

| **Parameter** |  |  |  |  |
| --- | --- | --- | --- | --- |
| **Table Analyzed** | **Data 1** |  |  |  |
| One-way analysis of variance |  |  |  |  |
| P value | 0.0002 |  |  |  |
| P value summary | *** |  |  |  |
| Are means signif. different? (P < 0.05) | Yes |  |  |  |
| Number of groups | 4 |  |  |  |
| F | 14.81 |  |  |  |
| R squared | 0.7737 |  |  |  |
| **ANOVA Table** | **SS** | **df** | **MS** |  |
| Treatment (between columns) | 259100 | 3 | 86350 |  |
| Residual (within columns) | 75780 | 13 | 5829 |  |
| Total | 334800 | 16 |  |  |
| **Newman-Keuls Multiple Comparison Test** | **Mean Diff.** | **q** | **Significant? P < 0.05?** | **Summary** |
| MEL vs MEL | -309.0 | 8.094 | Yes | *** |
| MEL vs DMSO | -190.0 | 5.247 | Yes | ** |
| MEL vs DMSO | -23.22 | 0.6083 | No | ns |
| DMSO vs MEL | -285.8 | 7.486 | Yes | *** |
| DMSO vs DMSO | -166.8 | 4.606 | Yes | ** |
| DMSO vs MEL | -119.0 | 3.285 | Yes | * |

|  | **DMSO** | **MEL** | **DMSO** | **MEL** |
| --- | --- | --- | --- | --- |
| **Number of values** | **4** | **4** | **5** | **4** |
| Minimum | 100.0 | 42.84 | 156.4 | 269.7 |
| 25% Percentile | 100.0 | 48.60 | 199.6 | 279.1 |
| Median | 100.0 | 77.87 | 306.0 | 350.6 |
| 75% Percentile | 100.0 | 103.9 | 314.4 | 527.6 |
| Maximum | 100.0 | 108.5 | 314.4 | 572.2 |
| Mean | 100.0 | 76.78 | 266.8 | 385.8 |
| Std. Deviation | 0.0 | 28.58 | 68.64 | 134.8 |
| Std. Error | 0.0 | 14.29 | 30.70 | 67.38 |
| Lower 95% CI | 100.0 | 31.30 | 181.6 | 171.3 |
| Upper 95% CI | 100.0 | 122.3 | 352.0 | 600.2 |
